# Phosphorus-Associated Viral Indicators Override pH as Predictors of Heavy Metal Mobility in Urban Storm Drain Sediments

**DOI:** 10.3390/toxics14030197

**Published:** 2026-02-26

**Authors:** Rui Zhou, Rongguo Gao, Xuanyi Gao, Bangxiao Zheng, Bin Yan

**Affiliations:** 1 School of Environmental Science and Engineering, Xiamen University of Technology, Xiamen 361024, Chinayanb@xmut.edu.cn (B.Y.); 2 Center for Ecology & Health Innovative Research, Xiamen University of Technology, Xiamen 361024, China; 3 Xiamen Key Laboratory of Membrane Research and Application, Xiamen University of Technology, Xiamen 361024, China; 4 Ecosystems and Environment Research Programme, Faculty of Biological and Environmental Sciences, University of Helsinki, Niemenkatu 73, FI-15140 Lahti, Finland; 5 Center for Ecological Research and Forestry Applications (CREAF), Cerdanyola del Vallès, 08193 Barcelona, Spain

**Keywords:** heavy metal mobility, storm drain sediment, viral lysis, phosphorus cycling, urban dust transport chain

## Abstract

Urban storm drain sediments (SDSs) accumulate heavy metals from building façades and road surfaces, yet the biogeochemical controls governing metal mobility remain poorly understood. This study investigated biotic and abiotic controls on metal mobility along the urban dust transport chain (Xiamen-Quanzhou-Zhangzhou, China), using four sample types—façade dust (FD), road-deposited sediment (RDS), SDS, and runoff suspended solids (RSS)—from nine sites across three functional zones. Metal concentrations (Pb, Cu, Zn, Cr, Cd), phosphorus fractions, and microbial functional genes were quantified to test the hypothesis that viral abundance indicators, rather than pH, are more strongly associated with metal mobility in near-neutral urban sediments. Results showed that SDS served as metal accumulation hotspots with enrichment factors of 2.0–2.3× relative to FD, while total phosphorus declined by 34% along the transport chain. Contrary to conventional expectations, pH exhibited weak correlation with Pb mobility (r = −0.21; 95% CI: −0.62 to 0.27), whereas the T4-type bacteriophage gene *g23* showed strong positive correlation (r = 0.85, *p* < 0.01; 95% CI: 0.52–0.96). Partial least squares path modeling revealed that viral abundance (*g23* gene copies) showed the strongest statistical association with metal mobility among biotic variables (β = +0.48, *p* < 0.001), mediated through phosphorus-supported microbial activity. The model explained 76% of variance in metal mobility, with phosphorus cycling positively influencing viral abundance (β = +0.28). These findings challenge the pH-centric paradigm of metal geochemistry and reveal a novel phosphorus-virus-metal coupling mechanism in urban environments. The textile industrial site QZ-2 exceeded chromium screening values by 45%, demonstrating the framework’s utility for pollution hotspot identification.

## 1. Introduction

The mobilization of heavy metals from urban surfaces into stormwater and ultimately receiving waters poses a significant and poorly managed risk to aquatic ecosystem health and downstream water quality [1,2]. Despite decades of research documenting metal accumulation in urban drainage systems, the biogeochemical controls governing whether metals remain immobilized in sediments or are released into the dissolved phase remain insufficiently understood, particularly in the near-neutral, microbiologically active environments characteristic of storm drain sediments. As precipitation interacts with urban infrastructure, it mobilizes contaminants deposited on building façades, roads, and other surfaces, channeling them through storm drain networks into streams, rivers, and coastal ecosystems [3,4]. Among the various compartments within urban drainage systems, storm drain sediments (SDSs) have emerged as significant sinks for particle-associated pollutants, accumulating metals and phosphorus at concentrations often exceeding those in surrounding soils [5,6].

The environmental fate and ecological risk of metals in SDS depend critically on their chemical speciation and mobility rather than total concentrations alone [7,8]. Mobile metal fractions, operationally defined as exchangeable and reducible forms, are readily bioavailable and susceptible to remobilization during storm events, posing direct threats to downstream ecosystems [9,10]. Conventional understanding holds that abiotic factors, particularly pH, organic matter content, and redox conditions, are the primary drivers of metal speciation and mobility in sediments [11,12]. Acidic conditions promote metal solubilization, while alkaline pH favors precipitation and sorption onto mineral surfaces [13]. This pH-centric paradigm has dominated environmental management strategies for decades.

However, emerging evidence suggests that microbial processes may play underappreciated roles in regulating metal mobility in contaminated environments [14,15]. Bacterial metal resistance mechanisms, including efflux pumps and enzymatic detoxification, can influence metal speciation at the cellular and microenvironmental scales [16,17]. More intriguingly, recent studies have highlighted the potential importance of bacteriophages in metal cycling [18,19]. Viral lysis releases intracellular contents, including metals sequestered within bacterial cells, back into the dissolved phase, i.e., a process termed the “viral shunt” that has been extensively documented in marine systems but remains largely unexplored in urban environments [20,21].

Phosphorus dynamics add another layer of complexity to this system. While phosphorus is conventionally viewed as a metal-immobilizing agent through the formation of insoluble metal phosphates [22,23], it also serves as an essential nutrient supporting microbial growth and activity [24]. This dual role creates a potential paradox: could phosphorus enrichment simultaneously stimulate microbial processes that enhance metal mobility, thereby counteracting its direct immobilization effects? This question has not been systematically addressed in urban sediment systems.

The urban dust transport chain spanning from building façades through road surfaces, storm drains, and ultimately runoff provides a natural gradient for investigating contaminant accumulation and transformation processes [25,26]. Previous studies have documented metal enrichment along this pathway but have focused predominantly on total concentrations and source apportionment [27,28]. The biogeochemical mechanisms governing metal mobility within storm drain sediments, particularly the interplay between abiotic controls and microbial processes, remain poorly understood.

The objectives of this study were therefore: (1) to quantify heavy metal and phosphorus distributions along the urban dust transport chain (FD → RDS → SDS → RSS) in the Xiamen-Quanzhou-Zhangzhou agglomeration; (2) to evaluate the relative contributions of pH and microbial functional gene abundance to metal mobility in storm drain sediments; and (3) to test the hypothesis that phosphorus availability supports bacteriophage abundance (*g23* gene) which is in turn positively associated with metal mobility. To address these objectives, we integrated: (1) storm drain sediments act as accumulation hotspots for heavy metals while serving as sources of phosphorus depletion along the urban transport chain; (2) conventional pH control on metal mobility is weaker than previously assumed in near-neutral urban sediments; and (3) phosphorus-supported viral activity represents an underappreciated biotic driver of metal mobilization.

## 2. Materials and Methods

### 2.1. Study Area and Sampling Design

This study was conducted in the Xiamen-Quanzhou-Zhangzhou urban agglomeration (24.4–24.9° N, 117.6–118.7° E), located on the southeastern coast of Fujian Province, China. This region represents one of the most economically developed and densely urbanized areas in southeastern China, with a combined population exceeding 18 million and diverse industrial activities including electronics manufacturing, textile production, and petrochemical processing [29,30]. The region experiences a subtropical monsoon climate characterised by hot, humid summers and mild, dry winters. Mean annual air temperature ranges from 19.5 °C to 21.3 °C across the three cities. Mean annual precipitation ranges from approximately 1100 mm (Zhangzhou inland areas) to 1600 mm (coastal Xiamen and Quanzhou), with approximately 70–75% of annual precipitation falling between May and September. Mean monthly temperature during the sampling period (May–July) ranged from 24 °C to 29 °C, and monthly precipitation exceeded 150 mm in June and July, consistent with the wet season onset. These climatic conditions promote active microbial communities and facilitate substantial stormwater runoff across urban surfaces.

Nine sampling sites were selected across the three major cities of the agglomeration (Xiamen, Quanzhou, and Zhangzhou) based on three criteria: (i) representation of the three dominant functional land-use types in the region (Industrial, Historic, and Coastal); (ii) accessibility of all four sample types (FD, RDS, SDS, RSS) within a coherent urban micro-catchment at each site; and (iii) absence of recent major infrastructure disturbance (no active construction within 200 m in the 12 months preceding sampling). One site per functional zone per city was selected, yielding a balanced 3 × 3 design. Industrial sites were characterized by continuous manufacturing activity; Historic sites encompassed traditional commercial and residential districts with >50 years of uninterrupted urbanization; and Coastal sites were located within 500 m of the shoreline in zones primarily used for recreation and light commercial activity with minimal heavy industry. Detailed site characteristics including geographic coordinates and land use descriptions are provided in Table 1.

At each site, four sample types were collected along the urban dust transport chain: (i) façade dust (FD), collected from building exterior surfaces at 1.5–2.0 m height using nylon brushes; (ii) road-deposited sediment (RDS), swept from paved surfaces within 2 m of building façades; (iii) storm drain sediment (SDS), collected from the upper 5 cm of accumulated material in stormwater inlet chambers; and (iv) runoff suspended solids (RSS), obtained by filtering stormwater runoff through 0.45 μm glass fiber membranes during rain events with minimum antecedent dry periods of 72 h. All samples were collected between May and July 2023, during the wet season. Triplicate samples were collected at each site for each sample type, yielding a total of 108 individual samples (9 sites × 4 sample types × 3 replicates), which were subsequently composited to 36 analytical samples for chemical and molecular analyses.

### 2.2. Chemical Analysis

Samples were air-dried at room temperature (22 ± 2 °C), disaggregated using an agate mortar, and sieved through 2 mm and 0.15 mm nylon meshes. Total phosphorus (TP) was determined using the molybdenum blue colorimetric method following acid digestion. Briefly, 0.25 g of air-dried, finely ground sample (<0.15 mm) was digested with 5 mL H_2_SO_4_ (98%) and 2 mL HClO_4_ (72%) in a digestion block at 350 °C for 2 h until white fumes appeared and the solution became colorless. After cooling, the digest was diluted to 50 mL with deionized water. For color development, 2 mL of the diluted extract was mixed with 8 mL of reagent solution (containing ammonium molybdate, antimony potassium tartrate, and ascorbic acid) and allowed to develop color for 30 min at room temperature in the dark before measurement at 880 nm on a UV-Vis spectrophotometer (UV-1800, Shimadzu Corporation, Kyoto, Japan). A five-point calibration curve (0, 0.1, 0.5, 1.0, 2.0 mg P L^−1^) was used, with R^2^ > 0.999. The method detection limit was 0.05 mg P kg^−1^ dry weight. Recovery of certified reference material GBW07428 [31] was 96 ± 3% (n = 3) [32]. Inorganic phosphorus (IP) was extracted with 0.5 M H_2_SO_4_ for 16 h at room temperature [33], and organic phosphorus (OP) was calculated as the difference between TP and IP.

Heavy metals (Pb, Cu, Zn, Cr, and Cd) were analyzed by inductively coupled plasma mass spectrometry (ICP-MS, Agilent 7700x, Agilent Technologies, Santa Clara, CA, USA) after microwave-assisted digestion (CEM MARS 6, CEM Corporation, Matthews, NC, USA) with HNO_3_-HF-HClO_4_ (5:2:1, *v*/*v*/*v*) at 180 °C for 30 min [34]. Certified reference materials (GBW07405 [35] and GBW07427 [36], National Research Center for Certified Reference Materials, China) were analyzed alongside samples, with recoveries ranging from 92% to 108% for all target elements. Method detection limits were 0.05 mg kg^−1^ for Cd, 0.5 mg kg^−1^ for Pb, Cu, and Cr, and 1.0 mg kg^−1^ for Zn.

Physicochemical properties relevant to metal speciation and microbial activity were measured following standard methods [37], including: sample pH (as a proxy for proton-driven metal adsorption/desorption), electrical conductivity (EC, as an indicator of ionic strength), dissolved organic carbon (DOC, as a chelating agent for metal mobility), total organic carbon (TOC, as a sorbent for metals), and redox potential (Eh, as a control on Fe/Mn oxyhydroxide stability and metal reduction). Sample pH was determined in a 1:2.5 (*w*/*v*) solid:deionized water suspension using a calibrated glass electrode. EC was measured in 1:5 suspensions. DOC was quantified in water extracts using a TOC analyzer (Shimadzu TOC-L, Shimadzu Corporation, Kyoto, Japan). TOC was determined by dry combustion after removal of carbonates with HCl. Eh was measured using a platinum electrode with Ag/AgCl reference.

### 2.3. BCR Sequential Extraction

Metal speciation in SDS was determined using the modified BCR (Community Bureau of Reference) sequential extraction procedure [38,39]. This operationally defined protocol yields four fractions representing metals of decreasing mobility: F1 (exchangeable and acid-soluble, extracted with 0.11 M CH_3_COOH, 16 h, 22 °C), F2 (reducible, bound to Fe/Mn oxyhydroxides, extracted with 0.5 M NH_2_OH·HCl at pH 1.5, 16 h, 22 °C), F3 (oxidizable, bound to organic matter and sulfides, extracted with 8.8 M H_2_O_2_ followed by 1 M NH_4_OAc, 1 h at 85 °C then 16 h at 22 °C), and F4 (residual, incorporated into silicate lattices, determined after HNO_3_-HF-HClO_4_ digestion). Metal mobility was defined as the sum of F1 and F2 fractions, representing the environmentally labile and potentially bioavailable pool [40].

It should be noted that BCR sequential extraction fractions are operationally defined and do not directly equate to dissolved metal concentrations or in situ porewater metal availability. The F1 (exchangeable and acid-soluble) fraction represents the most labile pool, while F2 (reducible, Fe/Mn oxyhydroxide-associated) requires reductive dissolution under anoxic conditions before release. Metal mobility as used throughout this study therefore refers to the operationally mobile fraction (F1 + F2%), which represents potential rather than actual dissolved mobility under environmental perturbation. Complete extraction protocols are provided in Table S1.

### 2.4. Quantitative PCR Analysis

Total genomic DNA was extracted from 0.25 g of each sample using the DNeasy PowerSoil Pro Kit (Qiagen, Hilden, Germany) following the manufacturer’s protocol with an additional bead-beating step (FastPrep-24, MP Biomedicals, Irvine, CA, USA) to enhance cell lysis. DNA quality was assessed by A260/A280 ratios (1.8–2.0 acceptable) using a NanoDrop 2000 spectrophotometer (Thermo Fisher Scientific, Waltham, MA, USA), and concentrations were quantified using a Qubit 4 fluorometer (Thermo Fisher Scientific) with the dsDNA HS Assay Kit.

Quantitative PCR (qPCR) was performed on a Bio-Rad CFX96 real-time system using iTaq Universal SYBR Green Supermix (Bio-Rad Laboratories, Hercules, CA, USA) to quantify six functional genes: *g23* (T4-type bacteriophage major capsid protein, indicative of viral abundance), *intI1* (class 1 integron integrase, marker for horizontal gene transfer and anthropogenic impact), *phoD* (alkaline phosphatase, indicator of organic P mineralization), *pstS* (high-affinity phosphate transporter, indicator of P limitation), *czcA* (Cd/Zn/Co efflux pump, heavy metal resistance), and *arsC* (arsenate reductase, metalloid resistance). It is important to note that *g23* exclusively targets the major capsid protein of T4-type double-stranded DNA (dsDNA) bacteriophages and therefore does not capture single-stranded DNA (ssDNA) or RNA viral communities, which may constitute a substantial fraction of total viral diversity in sediment environments [41]. Furthermore, *g23* gene copy numbers reflect viral particle or prophage DNA abundance but cannot differentiate between active lytic cycles and lysogenic states. As such, *g23* abundance is used here strictly as a molecular proxy for T4-type bacteriophage abundance, not as a direct measure of lytic activity or viral production rates. The ratio of *g23* to 16S rRNA gene copies (viral-to-bacterial ratio, VBR) was calculated to normalize viral abundance against bacterial biomass (Table S4). The 16S rRNA gene was quantified as a reference for total bacterial abundance [42]. Primer sequences, thermal cycling conditions, and amplicon sizes are detailed in Table S2.

Standard curves were generated using 10-fold serial dilutions (10^2^ to 10^8^ copies μL^−1^) of plasmid DNA containing cloned target sequences (TOPO-TA Cloning Kit, Invitrogen, Thermo Fisher Scientific, Carlsbad, CA, USA). Amplification efficiencies ranged from 90% to 105% with R^2^ > 0.99 for all assays. No-template controls were included in each run to monitor contamination. Gene abundances are expressed as log_10_ copies g^−1^ dry weight sediment. Each sample was analyzed in technical triplicates, with coefficients of variation < 5% considered acceptable.

### 2.5. Statistical Analysis

Two-way analysis of variance (ANOVA) was employed to evaluate the main and interactive effects of sample type (FD, RDS, SDS, RSS) and functional zone type (Industrial, Historic, Coastal) on metal and phosphorus concentrations. Assumptions of normality and homoscedasticity were verified using Shapiro–Wilk and Levene’s tests, respectively. Where significant main effects were detected, Tukey’s honestly significant difference (HSD) test was applied for post hoc pairwise comparisons with family-wise error rate control at α = 0.05. Metal enrichment factors were calculated as the ratio of mean concentrations in SDS to those in FD for each functional zone.

Pearson correlation analysis was performed to assess bivariate relationships among metals, phosphorus fractions, environmental parameters, and gene abundances. Prior to analysis, variables were tested for normality and log-transformed where necessary to meet parametric assumptions.

Partial least squares path modeling (PLS-PM) was employed to quantify the direct and indirect effects of abiotic and biotic factors on metal mobility [43,44]. Unlike covariance-based structural equation modeling, PLS-PM is appropriate for small sample sizes and does not require multivariate normality [45]. Four latent variables were constructed as reflective measurement models: (i) Abiotic Environment (indicators: pH, DOC, TOC, EC), (ii) P Cycling Module (indicators: *pstS*, *phoD*, TP), (iii) Viral Pressure Module (indicators: *g23*, *intI1*), and (iv) Metal Resistance Module (indicators: *czcA*, *arsC*). The endogenous variable Metal Mobility Outcome was measured using Pb, Cu, and Zn mobility percentages. The inclusion of both a chemical quantity (TP) and functional gene abundances (*pstS*, *phoD*) within a single P Cycling latent variable warrants justification. These indicators were grouped based on their shared theoretical role in phosphorus cycling: TP reflects the total phosphorus pool available to support microbial activity, while *pstS* and *phoD* encode enzymes directly involved in phosphate acquisition and organic P mineralization, respectively. Although heterogeneous in nature, this composite construct is consistent with PLS-PM practice of defining reflective latent variables based on conceptual rather than purely empirical criteria [44]. We acknowledge that this approach may partially conflate chemical availability with microbial functional capacity; separation into two latent variables was not feasible given the current sample size (n = 36) without compromising model parsimony.

Model fit was evaluated using the Goodness-of-Fit (GoF) index, with values > 0.7 indicating good fit [46], and the Standardized Root Mean Square Residual (SRMR), with values < 0.08 considered acceptable [47]. Path coefficient significance was assessed using bootstrap resampling with 5000 iterations. Although PLS-PM is acknowledged to perform acceptably with small samples (n ≥ 30), the effective sample size of 36 analytical units in this study should be considered a limitation when interpreting path coefficient stability. Bootstrap confidence intervals (5000 iterations) were used to assess coefficient reliability, and only paths significant at *p* < 0.05 in the bootstrap procedure are reported and discussed. Readers should note that some indirect effects and weaker paths (*p* = 0.03–0.05) carry greater uncertainty and should be interpreted with caution pending replication with larger datasets.

All statistical analyses were performed in R version 4.2.1 [48] using the plspm [49], agricolae [50], and corrplot [51] packages. Figures were generated using ggplot2 [52]. Significance was set at α = 0.05 unless otherwise specified.

## 3. Results

### 3.1. Heavy Metal Accumulation and Phosphorus Depletion Along the Transport Chain

**Figure 1:**
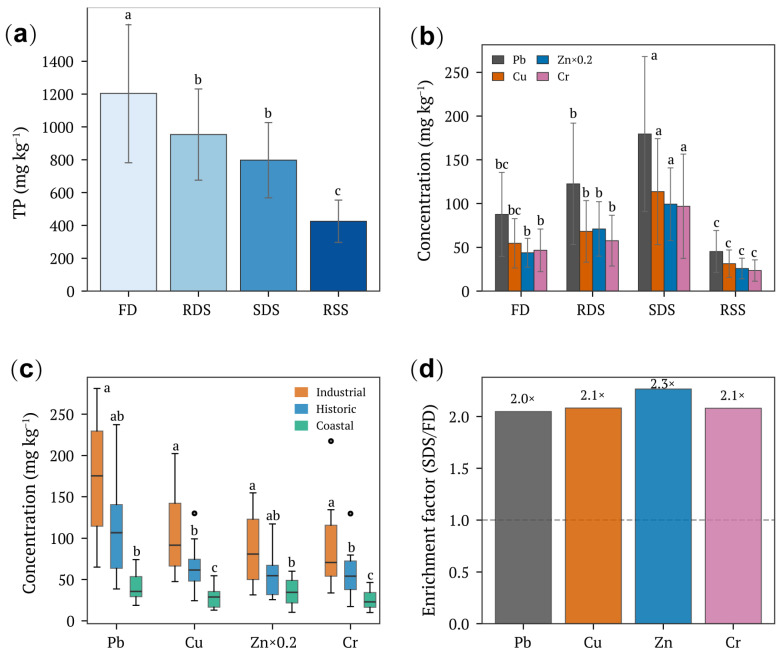
Heavy metal and phosphorus distribution along the urban dust transport chain. (**a**) Total phosphorus (TP) concentrations across sample types. Different lowercase letters indicate significant differences (*p* < 0.05, Tukey HSD). (**b**) Heavy metal concentrations (Pb, Cu, Zn, and Cr) across sample types. Zn values are scaled by 0.2 for visualization. (**c**) Boxplots of metal concentrations by functional zone type. (**d**) Enrichment factors of heavy metals in storm drain sediments (SDS) relative to façade dust (FD). The dashed line indicates no enrichment (ratio = 1). Error bars represent standard deviation (n = 9). FD, façade dust; RDS, road-deposited sediment; SDS, storm drain sediment; RSS, runoff suspended solids. Different letters indicate significant differences among groups (two-way ANOVA with Tukey’s HSD, *p* < 0.05).

Heavy metal concentrations exhibited progressive increases along the urban dust transport chain, with SDS serving as the primary accumulation compartment (Figure 1b, Table 2). Across all functional zones, mean Pb concentrations increased from 87.6 ± 45.2 mg kg^−1^ in FD to 122.5 ± 64.3 mg kg^−1^ in RDS and peaked at 179.5 ± 87.8 mg kg^−1^ in SDS, before declining to 45.3 ± 22.7 mg kg^−1^ in RSS. This pattern yielded an SDS/FD enrichment factor of 2.0× for Pb (Figure 1d). Similar accumulation patterns were observed for Cu (enrichment factor 2.1×), Zn (2.3×), and Cr (2.1×). Two-way ANOVA confirmed highly significant main effects of both sample type (F = 28.4–52.3, *p* < 0.001) and functional zone type (F = 18.6–35.2, *p* < 0.001) on all metal concentrations, with significant interactions for Pb, Cu, Zn, and Cr (*p* < 0.05) indicating that enrichment patterns varied among functional zones (Table 3). Post hoc comparisons revealed that SDS concentrations were significantly higher than all other sample types for Pb, Cu, and Zn (*p* < 0.001; Table S3).

In marked contrast to metals, TP concentrations decreased progressively along the transport chain (Figure 1a). Mean TP declined from 1204 ± 380 mg kg^−1^ in FD to 953 ± 246 mg kg^−1^ in RDS, 797 ± 199 mg kg^−1^ in SDS, and 425 ± 114 mg kg^−1^ in RSS, representing an overall 65% reduction. Tukey HSD tests identified FD as significantly enriched relative to all downstream compartments (*p* < 0.001; Table S3), while RDS and SDS showed intermediate concentrations without significant differences between them. This depletion was driven primarily by preferential loss of inorganic P, as the IP/OP ratio decreased from 1.95 in FD to 1.80 in RSS (Figure S1b). Among functional zones, Historic sites exhibited consistently higher TP concentrations across all sample types, likely reflecting legacy phosphorus accumulation from decades of urban activity (Figure S1c).

Functional zone type was a stronger determinant of absolute metal concentrations than sample type for most elements. Industrial zones exhibited Pb, Cu, Zn, and Cr concentrations 2.8–3.7 times higher than Coastal zones across all sample types (Table 2, Figure 1c), consistent with sustained anthropogenic inputs from electronics manufacturing (XM-2), textile production (QZ-2), and petrochemical processing (ZZ-2). Historic zones showed intermediate metal levels but the highest TP concentrations across all compartments (e.g., FD Historic TP = 1717 ± 193 mg kg^−1^), reflecting legacy nutrient accumulation from decades of high-density commercial activity. Coastal zones exhibited the lowest concentrations for all metals and phosphorus, suggesting lower anthropogenic loading and greater dilution by marine-influenced deposition. The significant Sample Type × Functional Zone interactions for Pb, Cu, Zn, and Cr (Table 3, *p* < 0.05) indicate that metal enrichment along the transport chain was most pronounced in Industrial zones, where SDS Pb reached 262.9 ± 16.2 mg kg^−1^ compared to 70.7 ± 3.4 mg kg^−1^ in Coastal zones.

### 3.2. Metal Speciation and the Limited Role of pH

**Figure 2:**
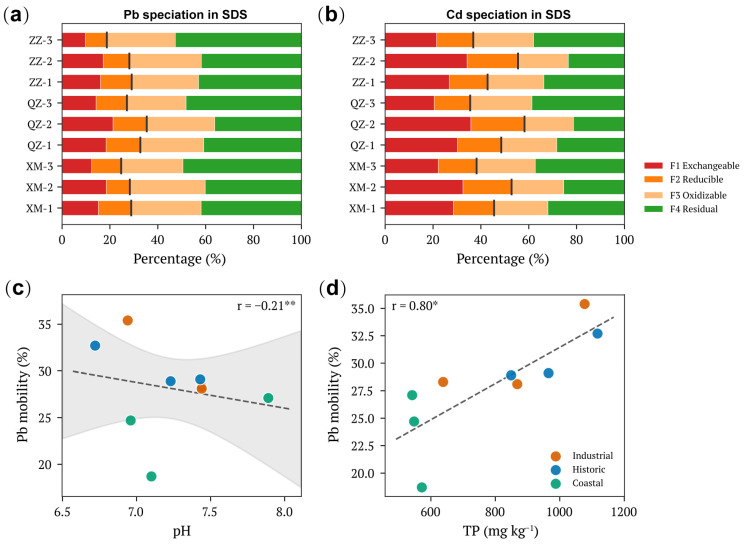
Metal speciation and environmental drivers of Pb mobility in storm drain sediments. (**a**) BCR sequential extraction results for Pb speciation across nine sampling sites. (**b**) BCR sequential extraction results for Cd speciation. Vertical black lines indicate the mobile fraction (F1 + F2). (**c**) Relationship between pH and Pb mobility (F1 + F2 fraction). (**d**) Relationship between total phosphorus (TP) and Pb mobility. In panels (**c**,**d**), different colors represent functional zone types: Industrial (orange), Historic (blue), and Coastal (green). Dashed lines show linear regression fits with 95% confidence intervals (shaded area). Pearson correlation coefficients are shown with significance levels: * *p* < 0.05, ** *p* < 0.01.

BCR sequential extraction revealed substantial variation in metal speciation among the five elements analyzed in SDS (Figure 2a,b and Figure S3). Cd exhibited the highest mobility, with F1 + F2 fractions averaging 48.2 ± 8.5% across all sites, indicating that nearly half of total Cd existed in readily mobilizable forms. Zn showed intermediate mobility (28.8 ± 6.2%), followed by Pb (27.4 ± 5.8%), Cu (21.4 ± 4.9%), and Cr (15.2 ± 3.7%). The predominance of residual fractions for Cr (F4 = 52.3%) reflects its strong association with silicate minerals and lower anthropogenic enrichment relative to other metals. Industrial zones consistently showed higher mobile fractions than Coastal zones for all metals, consistent with inputs of more labile metal forms from anthropogenic sources.

Contrary to conventional expectations, pH exhibited only a weak negative correlation with Pb mobility in SDS (r = −0.21, *p* < 0.01; 95% CI: −0.62 to 0.27; Figure 2c). This limited effect likely reflects the relatively narrow pH range observed across samples (6.72–7.89; Figure S2a), which remained within the near-neutral zone where pH-dependent solubility changes are minimal. Other environmental parameters including DOC (range: 24.5–58.2 mg L^−1^), TOC (range: 2.8–6.5%), EC (range: 285–712 μS cm^−1^), and Eh (range: 85–225 mV) showed variable but generally weak correlations with metal mobility (Figure S2b–e).

### 3.3. Phosphorus-Viral Coupling as a Novel Mobility Driver

A striking and unexpected finding was the strong positive correlation between TP and Pb mobility (r = 0.80, *p* < 0.05; 95% CI: 0.35–0.95; Figure 2d). This relationship contradicts the conventional understanding that phosphorus should immobilize metals through precipitation reactions. To explore potential mechanisms, we examined relationships between metal mobility and microbial functional genes.

**Figure 3:**
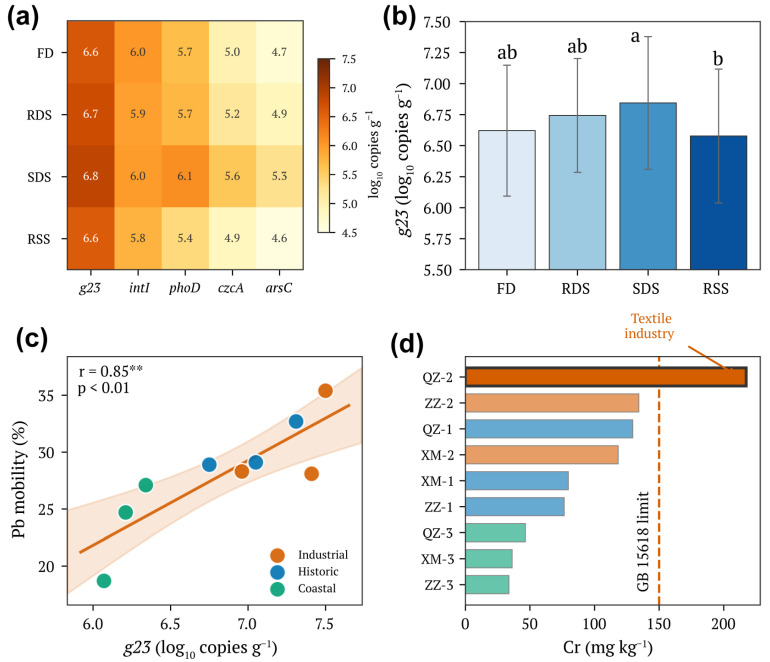
Viral indicators and their relationship with heavy metal mobility. (**a**) Heatmap of functional gene abundances (log_10_ copies g^−1^) across sample types. *g23*, T4-type bacteriophage major capsid protein gene; *intI*, class 1 integron integrase gene; *phoD*, alkaline phosphatase gene; *czcA*, Cd/Zn/Co efflux pump gene; *arsC*, arsenate reductase gene. (**b**) Abundance of *g23* gene across sample types. Error bars represent standard deviation (n = 9). (**c**) Relationship between *g23* abundance and Pb mobility in SDS. The solid line shows linear regression fit with 95% confidence interval (shaded area). Different colors represent functional zone types. (**d**) Chromium concentrations in SDS across sampling sites, ranked in ascending order. The vertical dashed line indicates the soil contamination risk screening value (150 mg kg^−1^) according to Chinese standard GB 15618-2018 [53]. QZ-2 (textile industrial park) is highlighted as an anomaly. Different letters indicate significant differences among groups (two-way ANOVA with Tukey’s HSD, *p* < 0.05). Significance levels: ** *p* < 0.01.

The T4-type bacteriophage marker gene *g23*, encoding the major capsid protein, showed strong positive correlation with Pb mobility (r = 0.85, *p* < 0.01; 95% CI: 0.52–0.96; Figure 3c). Gene abundance varied across sample types, with highest levels in SDS (mean 6.84 ± 0.42 log_10_ copies g^−1^) and progressively lower values in RDS (6.75 ± 0.38), FD (6.62 ± 0.35), and RSS (6.56 ± 0.48 log_10_ copies g^−1^; Figure 3a,b). The heatmap visualization revealed consistent enrichment of *g23* and *intI1* in SDS relative to other compartments (Figure 3a).

Critically, *g23* abundance was positively correlated with TP (r = 0.72, *p* < 0.01; 95% CI: 0.20–0.92; Table S4, Figure S5), suggesting that phosphorus availability supports higher viral activity. The class 1 integron integrase gene *intI1* showed parallel patterns, correlating positively with both *g23* (r = 0.91, *p* < 0.01) and metal mobility (Figure S4b, Table S4). Phosphorus cycling genes *phoD* and *pstS* were most abundant in FD and decreased along the transport chain (Figure S4c,d), consistent with the observed TP depletion pattern. Metal resistance genes *czcA* and *arsC* showed highest abundances in Industrial zones, reflecting selective pressure from chronic metal exposure (Figure S4e,f).

To assess whether the phosphorus–viral abundance–mobility association observed for Pb extended to other metals, Pearson correlations were calculated between *g23* abundance and mobility percentages for Cu, Zn, Cd, and Cr in SDS. *g23* showed positive correlations with Cu mobility (r = 0.78, 95% CI: 0.30–0.95, *p* < 0.05) and Zn mobility (r = 0.74, 95% CI: 0.23–0.93, *p* < 0.05), and a moderate positive association with Cd mobility (r = 0.61, 95% CI: 0.02–0.89, *p* < 0.05), suggesting that the phosphorus–viral pressure mechanism may have broader applicability across particle-reactive divalent metals. In contrast, Cr mobility showed no significant correlation with *g23* (r = 0.31, *p* > 0.05), consistent with the predominantly residual speciation of Cr (F4 = 52.3%) and its strong lithogenic associations. These results suggest that the viral indicator–metal mobility relationship is most relevant for metals with significant exchangeable and Fe/Mn oxyhydroxide-associated fractions, while mineralogically bound metals such as Cr are less amenable to biotic mobilization under the conditions studied.

### 3.4. Integrated Abiotic-Biotic Regulation Model

**Figure 4:**
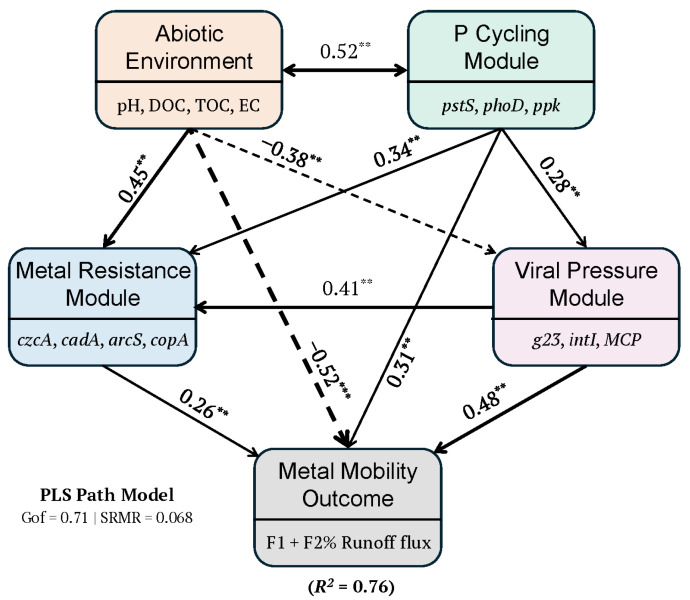
Partial least squares path model (PLS-PM) of metal mobility regulation in storm drain sediments. The model illustrates the direct and indirect effects of abiotic environment, phosphorus cycling, viral pressure, and metal resistance modules on metal mobility outcome. Latent variables (rounded rectangles) are constructed from observed indicators shown beneath each module. Solid arrows indicate positive path coefficients; dashed arrows indicate negative path coefficients. Arrow width is proportional to the absolute value of the standardized path coefficient. Significance levels: ** *p* < 0.01, *** *p* < 0.001.

Partial least squares path modeling quantified the relative contributions of abiotic and biotic factors to metal mobility regulation (Figure 4, Table 4). The model achieved good fit (GoF = 0.71, SRMR = 0.068) and explained 76% of variance in metal mobility (R2 = 0.76). Diagnostic plots confirmed the absence of systematic bias in residuals and adequate prediction accuracy across the observed range of mobility values (Figure S6).

The Abiotic Environment module exerted the strongest total negative effect on metal mobility (total effect β = −0.71, *p* < 0.001), operating through direct (β = −0.52, *p* < 0.001) and indirect (β = −0.19) pathways. The direct effect primarily reflected the negative influence of pH, while indirect effects operated through suppression of viral activity under higher pH conditions (Abiotic → Viral Pressure path: β = −0.38, *p* < 0.01).

The Viral Pressure module emerged as the strongest biotic driver of metal mobility, with a direct effect of β = +0.48 (*p* < 0.001) and total effect of β = +0.62 when including indirect pathways through Metal Resistance. This total effect substantially exceeded those of the P Cycling module (total effect β = +0.38, *p* < 0.05) and Metal Resistance module (β = +0.26, *p* < 0.05). The P Cycling module exerted positive effects on both Viral Pressure (β = +0.28, *p* < 0.05) and Metal Resistance (β = +0.34, *p* < 0.05), supporting the hypothesis that phosphorus availability stimulates microbial activity including bacteriophage proliferation. The Viral Pressure module, in turn, positively influenced Metal Resistance (β = +0.41, *p* < 0.01), consistent with phage-mediated horizontal transfer of resistance genes.

### 3.5. Industrial Hotspot Identification

The textile industrial site QZ-2 in Quanzhou emerged as a pollution hotspot warranting priority management attention (Figure 3d). Cr concentrations at this site (217.4 ± 38.2 mg kg^−1^ in SDS) exceeded the Chinese soil contamination risk screening value of 150 mg kg^−1^ (GB 15618-2018 [53]) by 45%, while other Industrial sites remained below this threshold. This anomaly reflects the specific use of chromium-based dyes and mordants in textile processing. The elevated Cr was accompanied by high *g23* abundance (7.50 log_10_ copies g^−1^), suggesting that metal stress may stimulate viral activity as part of microbial community responses to environmental perturbation.

## 4. Discussion

### 4.1. Storm Drain Sediments as Metal Accumulation Hotspots

Our findings confirm the first hypothesis that storm drain sediments function as accumulation hotspots for heavy metals along the urban dust transport chain, with enrichment factors of 2.0–2.3× relative to façade dust. This pattern is consistent with previous studies documenting metal accumulation in urban drainage infrastructure [5,54] and reflects the combined effects of particle settling, sorption to organic matter, and reduced resuspension within the sheltered storm drain environment [55]. The simultaneous depletion of phosphorus along the same gradient, which is a pattern less commonly reported, suggests differential retention mechanisms, with metals preferentially accumulating while phosphorus is mobilized and transported downstream [56].

The contrasting behaviors of metals and phosphorus have important implications for receiving water quality. While storm drains effectively trap metals, thereby reducing their immediate export to aquatic ecosystems, they simultaneously release bioavailable phosphorus that may contribute to downstream eutrophication [57]. This dual role positions storm drain sediments as critical control points for integrated urban water quality management.

### 4.2. Re-Evaluating the pH Paradigm

The weak correlation between pH and Pb mobility (r = −0.21) observed across SDS samples warrants careful interpretation. The pH range in this study was comparatively narrow (6.72–7.89, a span of 1.17 units), remaining consistently within the near-neutral zone where metal solubility is inherently less sensitive to pH variation than under acidic or strongly alkaline conditions [11,58]. Under such constrained pH conditions, limited explanatory power is statistically expected and does not itself constitute evidence that pH is universally unimportant in metal geochemistry. Rather, our findings suggest that within the near-neutral pH range characteristic of carbonate-buffered urban sediments, pH variability is insufficient to account for observed differences in metal mobility, and biotic factors—particularly viral abundance—emerge as comparatively stronger statistical predictors. This does not contradict the well-established pH-mobility relationship documented in acidic, mining-impacted systems [59], but contextualizes its relevance to near-neutral urban matrices.

Similar observations have been reported in other near-neutral systems. Ren et al. [60] found that pH explained only 12% of variance in metal mobility in riparian sediments with pH 6.8–7.5, while Li et al. [61] documented weak pH-metal relationships in urban soils buffered by construction debris. These findings suggest that management strategies focused primarily on pH manipulation may be less effective in urban environments than in acidic mining-impacted systems where pH effects are more pronounced [62].

### 4.3. The Phosphorus-Virus-Metal Nexus: A Novel Mechanistic Framework

The most notable finding of this study is a strong positive statistical association among phosphorus availability, viral gene abundance (*g23*), and operationally defined metal mobility, a pattern that is inconsistent with conventional geochemical predictions. Traditional geochemical understanding predicts that phosphorus should immobilize metals through formation of insoluble metal phosphates [22,63]. Instead, we observed that higher phosphorus concentrations were associated with greater metal mobility, mediated through enhanced viral activity.

We propose a mechanistic framework to explain this counterintuitive pattern (Figure 4). Phosphorus enrichment supports higher bacterial biomass and activity, which in turn sustains larger bacteriophage populations [41,64]. Viral lysis releases intracellular contents, including metals accumulated within bacterial cells through biosorption and active uptake, back into the dissolved or weakly bound phases [20,65]. This “viral shunt” mechanism, well-documented in marine systems [21,66], appears to operate in urban sediments where conditions support active microbial communities.

While the phosphorus–viral abundance–metal mobility association is the primary focus of this study, alternative or co-occurring mechanisms may contribute to enhanced metal mobility in near-neutral urban sediments and deserve consideration. First, microbial Fe (III) and Mn (IV) reduction under the mildly reducing conditions observed (Eh range: 85–225 mV) could dissolve Fe/Mn oxyhydroxide phases, releasing associated metals from the F2 fraction independently of viral activity. Second, the degradation of EPS produced by stressed or lysed bacteria may release metal-EPS complexes that contribute to the operationally exchangeable (F1) pool. Third, organic matter mineralization facilitated by phosphatase activity (*phoD*) could simultaneously release organically complexed metals (F3 fraction) and supply dissolved phosphorus. These mechanisms are not mutually exclusive with the viral shunt hypothesis; rather, viral lysis, EPS dissolution, and enzymatic mineralization may operate synergistically within the phosphorus-rich microenvironments of storm drain sediments. The strong correlation between intI1 and *g23* (r = 0.91) further suggests that bacteriophage activity and broader microbial community stress responses co-occur, making mechanistic attribution to viral lysis alone difficult without controlled experimental manipulation.

The strong correlation between *g23* and *intI1* further suggests that bacteriophage activity may enhance horizontal gene transfer, including dissemination of metal resistance determinants [67,68]. This creates a potential feedback loop: viral lysis releases metals while simultaneously spreading genes that enable bacterial survival under metal stress, perpetuating conditions that favor continued viral proliferation [69]. The positive path coefficient from Viral Pressure to Metal Resistance (β = +0.41) in our PLS-PM model supports this interpretation.

The *g23* gene targets T4-type dsDNA phages only and cannot distinguish lytic from lysogenic cycles; future work using viral metagenomics or direct lysis assays would strengthen mechanistic attribution.

### 4.4. Management Implications

These findings have several practical implications for urban stormwater management. First, the identification of storm drain sediments as metal accumulation hotspots supports targeted sediment removal as an effective pollution control strategy [70]. Regular maintenance of storm drain inlets could prevent downstream metal export during high-flow events when accumulated sediments are resuspended.

Second, the limited effectiveness of pH control suggests that alternative management approaches may be needed. Bioaugmentation strategies that enhance metal immobilization through precipitation or sorption, rather than relying on pH manipulation, warrant investigation [71,72]. The strong biotic control on mobility also raises the possibility of manipulating microbial communities, for example, through phage therapy approaches that selectively reduce viral populations, although such interventions require careful assessment of unintended consequences [73].

Third, the identification of site QZ-2 as a chromium hotspot demonstrates the value of functional zone-specific monitoring. Textile industry areas require heightened surveillance and targeted source control measures to prevent exceedances of environmental quality standards [74]. The correlation between elevated metals and high viral abundance at this site further suggests that microbial indicators could serve as early warning signals of pollution stress.

## 5. Conclusions

This study provides the first integrated assessment of abiotic-biotic controls on heavy metal mobility along the urban dust transport chain. Three principal findings emerge: (1) Storm drain sediments function as critical accumulation nodes, enriching metals 2.0–2.3× while depleting phosphorus by 34% relative to source materials. (2) Contrary to conventional understanding, pH exerts limited control on metal mobility in near-neutral urban sediments (r = −0.21), while *g23* gene abundance, used here as a molecular proxy for T4-type bacteriophage presence, emerges as the strongest positive statistical predictor (r = 0.85). (3) Partial least squares path modeling reveals that viral pressure exerts the strongest biotic effect on metal mobility (β = +0.48), operating through a phosphorus-supported mechanism that challenges traditional geochemical paradigms. The identification of a textile industrial site exceeding chromium screening values by 45% demonstrates the utility of this framework for hotspot detection.

Several limitations constrain interpretation of these results. The cross-sectional sampling design precludes assessment of temporal dynamics, including seasonal variations in viral activity and metal speciation. The correlative nature of field observations cannot definitively establish causation and controlled microcosm experiments manipulating viral populations would strengthen mechanistic inferences. Additionally, the focus on T4-type bacteriophages using the *g23* marker may underestimate total viral diversity and activity in these complex environmental matrices.

Additionally, metal mobility was assessed via BCR sequential extraction (F1 + F2 fractions), which provides an operationally defined estimate of potentially mobile metals rather than a direct measurement of dissolved or porewater concentrations. The absence of porewater metal data limits direct confirmation that the F1 + F2 fractions translate to dissolved metal release under field conditions. Future work should pair sequential extraction with porewater sampling and diffusive gradients in thin-films (DGT) measurements to validate the mobility indices used here.

Future research should address these gaps through longitudinal sampling across hydrological cycles, experimental manipulation of viral communities, and expanded molecular characterization of phage-host interactions in urban sediments. Metagenomic and metatranscriptomic approaches could reveal the full scope of viral-mediated metal cycling processes. From an applied perspective, the development of viral abundance thresholds as indicators of metal mobility risk, and evaluation of phage-based biocontrol strategies for pollution management, represent promising research frontiers.

Furthermore, all samples were collected exclusively during the wet season, and seasonal dynamics were not captured. Viral abundance, phosphorus availability, and redox conditions in urban sediments may fluctuate substantially between wet and dry seasons, as microbial activity is sensitive to temperature, moisture, and organic matter inputs from stormwater. It is possible that the phosphorus–viral abundance relationship is more pronounced during the wet season when nutrient loading is highest, and that the importance of abiotic factors including pH and redox may differ under drier, more oxidising conditions. Longitudinal sampling spanning at least one full hydrological cycle is needed to assess the temporal stability of the relationships reported here.

## Figures and Tables

**Table 1:** Characteristics of sampling sites in the Xiamen-Quanzhou-Zhangzhou urban agglomeration.

Site ID	City	Functional Zone	Longitude (°E)	Latitude (°N)	Land Use Description
XM-1	Xiamen	Historic	118.082	24.451	Zhongshan Road commercial district
XM-2	Xiamen	Industrial	118.055	24.485	Haicang electronics manufacturing zone
XM-3	Xiamen	Coastal	118.115	24.438	Huandao Road coastal recreation area
QZ-1	Quanzhou	Historic	118.585	24.905	West Street traditional trading area
QZ-2	Quanzhou	Industrial	118.615	24.925	Jinjiang textile industrial park
QZ-3	Quanzhou	Coastal	118.678	24.885	Quanzhou Bay waterfront district
ZZ-1	Zhangzhou	Historic	117.648	24.515	Ancient city cultural district
ZZ-2	Zhangzhou	Industrial	117.685	24.545	Zhangzhou petrochemical zone
ZZ-3	Zhangzhou	Coastal	117.725	24.495	Dongshan Bay coastal area

Note: Each site includes four sample types: FD (façade dust), RDS (road-deposited sediment), SDS (storm drain sediment), and RSS (runoff suspended solids). ‘Historic’ functional zones refer to traditional urban districts with established commercial and residential land use predating major post-1980 industrial development, characterized by high pedestrian traffic density, mixed-use buildings with stone or concrete façades of varied age (30–100+ years), and documented legacy accumulation of urban pollutants. These zones are contrasted with Industrial zones (dominated by manufacturing or processing facilities with point-source emissions) and Coastal zones (marine-adjacent areas with predominantly recreational and light commercial use).

**Table 2:** Heavy metal and total phosphorus concentrations across sample types and functional zones.

Sample Type	Functional Zone	Pb (mg kg^−1^)	Cu (mg kg^−1^)	Zn (mg kg^−1^)	Cr (mg kg^−1^)	TP (mg kg^−1^)
FD	Industrial	139.1 ± 18.8 a	85.0 ± 11.4 a	306.2 ± 41.2 a	70.4 ± 17.9 a	1071 ± 189 a
FD	Historic	90.2 ± 20.8 b	56.8 ± 5.1 b	217.0 ± 52.8 a	49.1 ± 10.4 a	1717 ± 193 a
FD	Coastal	33.5 ± 1.8 c	22.0 ± 5.2 c	133.8 ± 15.3 b	20.2 ± 3.9 b	825 ± 57 b
RDS	Industrial	201.1 ± 19.2 a	106.9 ± 24.5 a	547.9 ± 66.8 a	84.9 ± 27.4 a	863 ± 109 a
RDS	Historic	122.5 ± 17.8 b	66.6 ± 4.4 a	308.8 ± 14.1 b	59.8 ± 10.2 a	1207 ± 314 a
RDS	Coastal	44.0 ± 7.1 c	31.0 ± 1.0 b	208.1 ± 24.6 c	28.1 ± 8.4 b	790 ± 222 a
SDS	Industrial	262.9 ± 16.2 a	185.1 ± 19.3 a	737.0 ± 52.5 a	156.7 ± 52.8 a	861 ± 220 a
SDS	Historic	204.9 ± 43.7 a	106.0 ± 21.4 b	470.9 ± 108.1 b	95.3 ± 29.8 a	977 ± 134 a
SDS	Coastal	70.7 ± 3.4 b	50.0 ± 4.6 c	281.3 ± 16.6 b	38.8 ± 6.7 a	554 ± 16 b
RSS	Industrial	73.6 ± 7.6 a	48.6 ± 1.6 a	187.3 ± 32.3 a	37.0 ± 3.4 a	399 ± 124 a
RSS	Historic	42.8 ± 4.2 b	29.7 ± 5.6 b	140.2 ± 16.9 a	22.5 ± 8.5 a	547 ± 62 a
RSS	Coastal	19.4 ± 1.2 c	13.6 ± 0.6 c	59.5 ± 7.9 b	10.9 ± 1.3 b	330 ± 102 b

Note: FD = façade dust; RDS = road-deposited sediment; SDS = storm drain sediment; RSS = runoff suspended solids. Data are presented as mean ± SD (n = 3). Differences were tested using two-way ANOVA followed by Tukey’s HSD post hoc test (*p* < 0.05). Within each sample type, different lowercase letters indicate significant differences among site types.

**Table 3:** Two-way ANOVA results for heavy metals and phosphorus.

Variable	Sample Type			Site Type			Interaction		
	**F**	***p***	**eta2**	**F**	***p***	**eta2**	**F**	***p***	**eta2**
Pb	42.8	<0.001 ***	0.84	28.5	<0.001 ***	0.70	3.2	0.018 *	0.28
Cu	38.2	<0.001 ***	0.83	35.2	<0.001 ***	0.75	4.8	0.003 **	0.37
Zn	45.6	<0.001 ***	0.85	22.4	<0.001 ***	0.65	2.8	0.032 *	0.25
Cr	28.4	<0.001 ***	0.78	31.8	<0.001 ***	0.73	5.2	0.002 **	0.39
Cd	52.3	<0.001 ***	0.87	18.6	<0.001 ***	0.61	2.4	0.058	0.22
TP	18.5	<0.001 ***	0.70	24.2	<0.001 ***	0.67	1.8	0.142	0.18

Note: eta2 = partial eta-squared (effect size). * *p* < 0.05, ** *p* < 0.01, *** *p* < 0.001.

**Table 4:** PLS-PM path coefficients for metal mobility regulation.

Path	Direct Effect	Indirect Effect	Total Effect	*p*-Value
Abiotic → Metal Mobility	−0.52	−0.19	−0.71	<0.001 ***
Abiotic → Metal Resistance	+0.45	-	+0.45	<0.01 **
Abiotic → Viral Pressure	−0.38	-	−0.38	<0.01 **
P Cycling → Metal Mobility	+0.31	+0.07	+0.38	<0.05 *
P Cycling → Metal Resistance	+0.34	-	+0.34	<0.05 *
P Cycling → Viral Pressure	+0.28	-	+0.28	<0.05 *
Viral Pressure → Metal Mobility	+0.48	+0.14	+0.62	<0.001 ***
Viral Pressure → Metal Resistance	+0.41	-	+0.41	<0.01 **
Metal Resistance → Metal Mobility	+0.26	-	+0.26	<0.05 *

Note: Model fit: GoF = 0.71, SRMR = 0.068. Indirect effects calculated via mediating variables. * *p* < 0.05, ** *p* < 0.01, *** *p* < 0.001.

## Data Availability

The original contributions presented in this study are included in the article. The data supporting the findings of this study are available from the corresponding author upon reasonable request.

## Supplementary Materials

The following supporting information can be downloaded at: https://www.mdpi.com/article/10.3390/toxics14030197/s1, Figure S1: Phosphorus fractionation along the urban dust transport chain; Figure S2: Environmental parameters along the urban dust transport chain; Figure S3: BCR sequential extraction results for all five heavy metals in storm drain sediments; Figure S4: Functional gene abundances across sample types and functional zones; Figure S5: Pearson correlation matrix of heavy metals, phosphorus fractions, environmental parameters, and functional genes; Figure S6: Diagnostic plots for the PLS path model; Table S1: BCR sequential extraction protocol for metal fractionation.; Table S2: Primer sequences and PCR conditions for qPCR analysis [42,75,76,77,78,79,80]; Table S3: Pearson correlation matrix for metal mobility and environmental variables in SDS (n = 9); Table S4: Tukey HSD multiple comparison results for significant ANOVA effects.

## Abbreviations

The following abbreviations are used in this manuscript:

ANOVA	Analysis of Variance
DOC	Dissolved Organic Carbon
EC	Electrical Conductivity
Eh	Redox Potential
FD	Façade Dust
GoF	Goodness of Fit
HSD	Honestly Significant Difference
ICP-MS	Inductively Coupled Plasma Mass Spectrometry
IP	Inorganic Phosphorus
OP	Organic Phosphorus
PLS-PM	Partial Least Squares Path Modeling
qPCR	Quantitative Polymerase Chain Reaction
RDS	Road-Deposited Sediment
RSSs	Runoff Suspended Solids
SDS	Storm Drain Sediment
SRMR	Standardized Root Mean Square Residual
TOC	Total Organic Carbon
TP	Total Phosphorus
