# Supplementary material for: Phosphorus-Associated Viral Indicators Override pH as Predictors of Heavy Metal Mobility in Urban Storm Drain Sediments
Source: Toxics. 2026 Feb 26;14(3):197. doi: 10.3390/toxics14030197 (PMC13030558; doi:10.3390/toxics14030197)
Supplement: Supplementary file 1 [file toxics-14-00197-s001.zip › toxics-4146238-supplementary.pdf]

toxics

MDPI

Supplementary Information for

Phosphorus-Associated Viral Indicators Overrides pH as Predictors of Heavy Metal Mobility in Urban Storm Drain Sediments

Rui Zhou [1,2#], Rongguo Gao [1#], Xuanyi Gao[1], Bangxiao Zheng[2,3,4,5*], Bin Yan[1,3]

1 School of Environmental Science and Engineering, Xiamen University of Technology, Xiamen 361024, P. R. China;
2 Center for Ecology & Health Innovative Research, Xiamen University of Technology, Xiamen 361024, P. R. China;
3 Xiamen Key Laboratory of Membrane Research and Application, Xiamen 361024, P. R. China;
4 Faculty of Biological and Environmental Sciences, Ecosystems and Environment Research Programme, Niemenkatu 73, FI-15140, Lahti, University of Helsinki, Finland;
5 CREAF, Cerdanyola del Vallès, 08193 Barcelona, Catalonia, Spain;
* Correspondence: bangxiao.zheng@xmut.edu.cn; Tel.: (+86) 13774659187

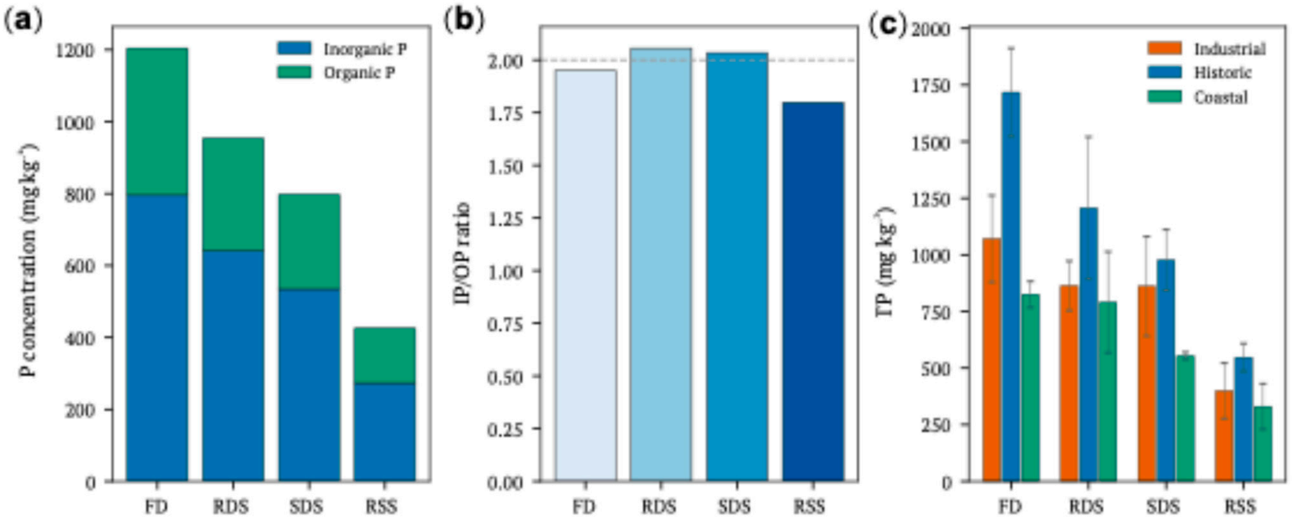

Figure S1. Phosphorus fractionation along the urban dust transport chain. (a) Stacked bar chart showing inorganic phosphorus (IP) and organic phosphorus (OP) concentrations across sample types. (b) IP/OP ratio across sample types. The dashed line indicates a ratio of 2.0. (c) Total phosphorus (TP) concentrations grouped by functional zone type within each sample type. Error bars represent standard deviation (n = 3). FD, façade dust; RDS, road-deposited sediment; SDS, storm drain sediment; RSS, runoff suspended solids.

https://doi.org/10.3390/toxics14030197

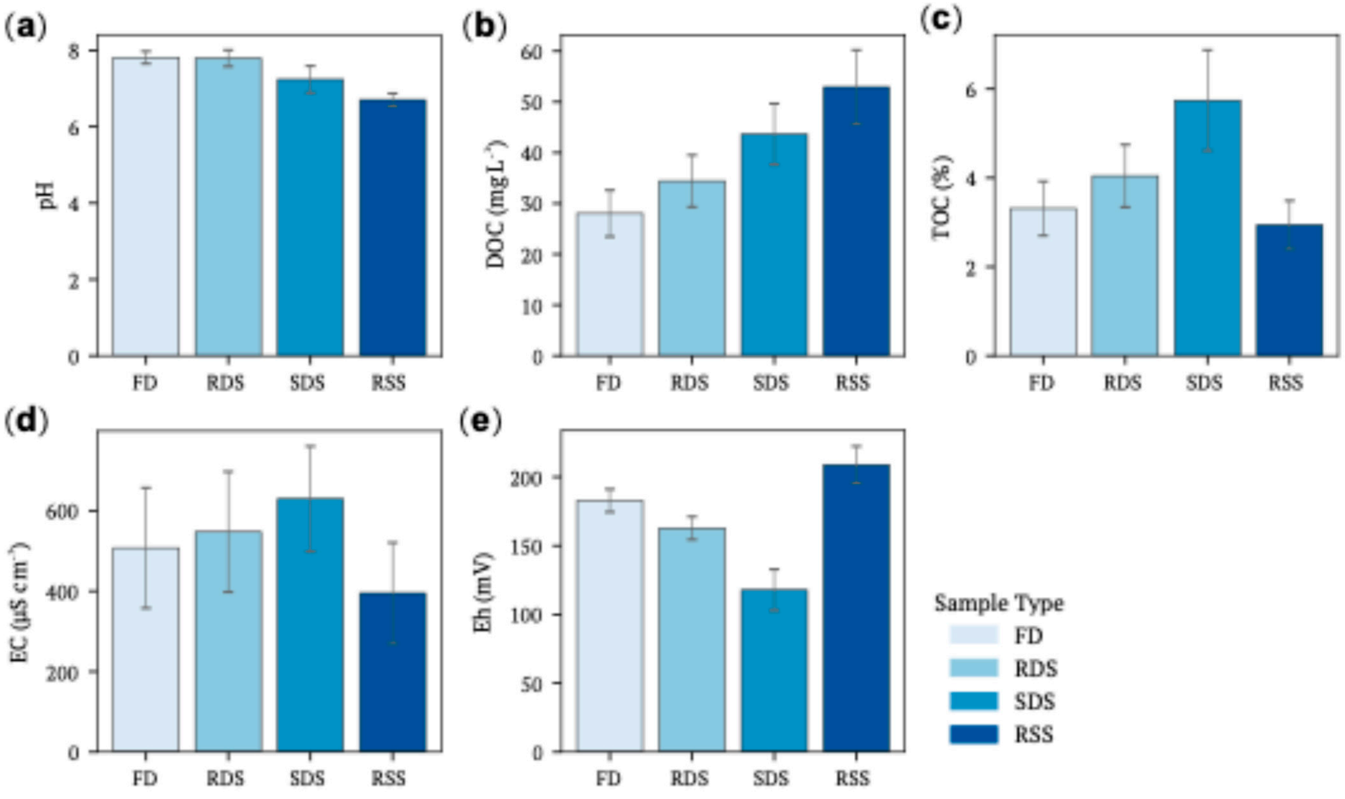

Figure S2. Environmental parameters along the urban dust transport chain. (a) pH, (b) dissolved organic carbon (DOC), (c) total organic carbon (TOC), (d) electrical conductivity (EC), and (e) redox potential (Eh) across sample types. Error bars represent standard deviation (n = 9). Different colors represent sample types along the transport chain from façade dust (FD) to runoff suspended solids (RSS).

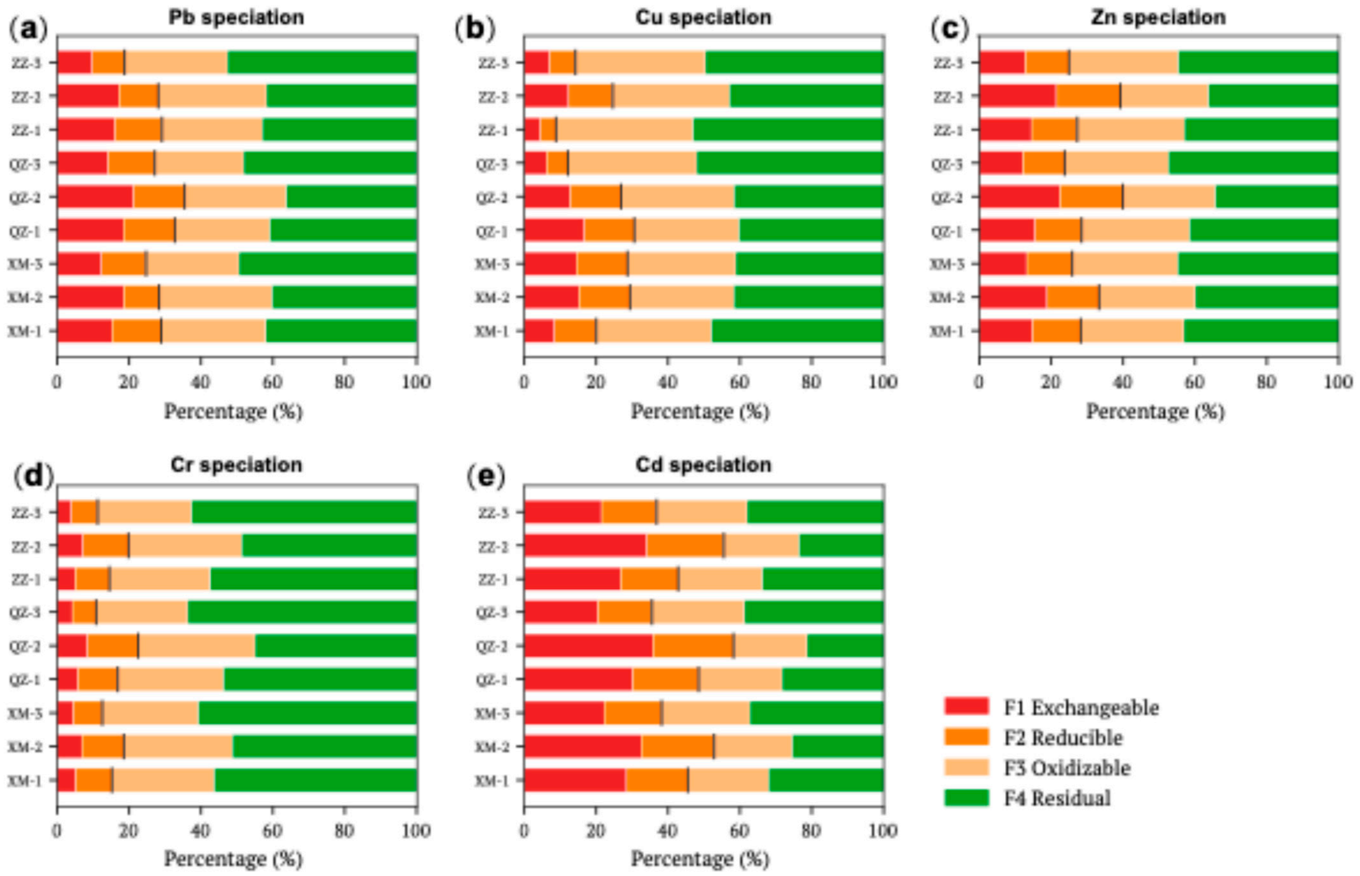

Figure S3. BCR sequential extraction results for all five heavy metals in storm drain sediments. Percentage distribution of (a) Pb, (b) Cu, (c) Zn, (d) Cr, and (e) Cd among four BCR fractions across nine sampling sites. F1, exchangeable fraction (acid-soluble); F2, reducible fraction (bound to Fe/Mn oxides); F3, oxidizable fraction (bound to organic matter and sulfides); F4, residual fraction (bound to silicate minerals). Vertical black lines indicate the mobile fraction (F1 + F2).

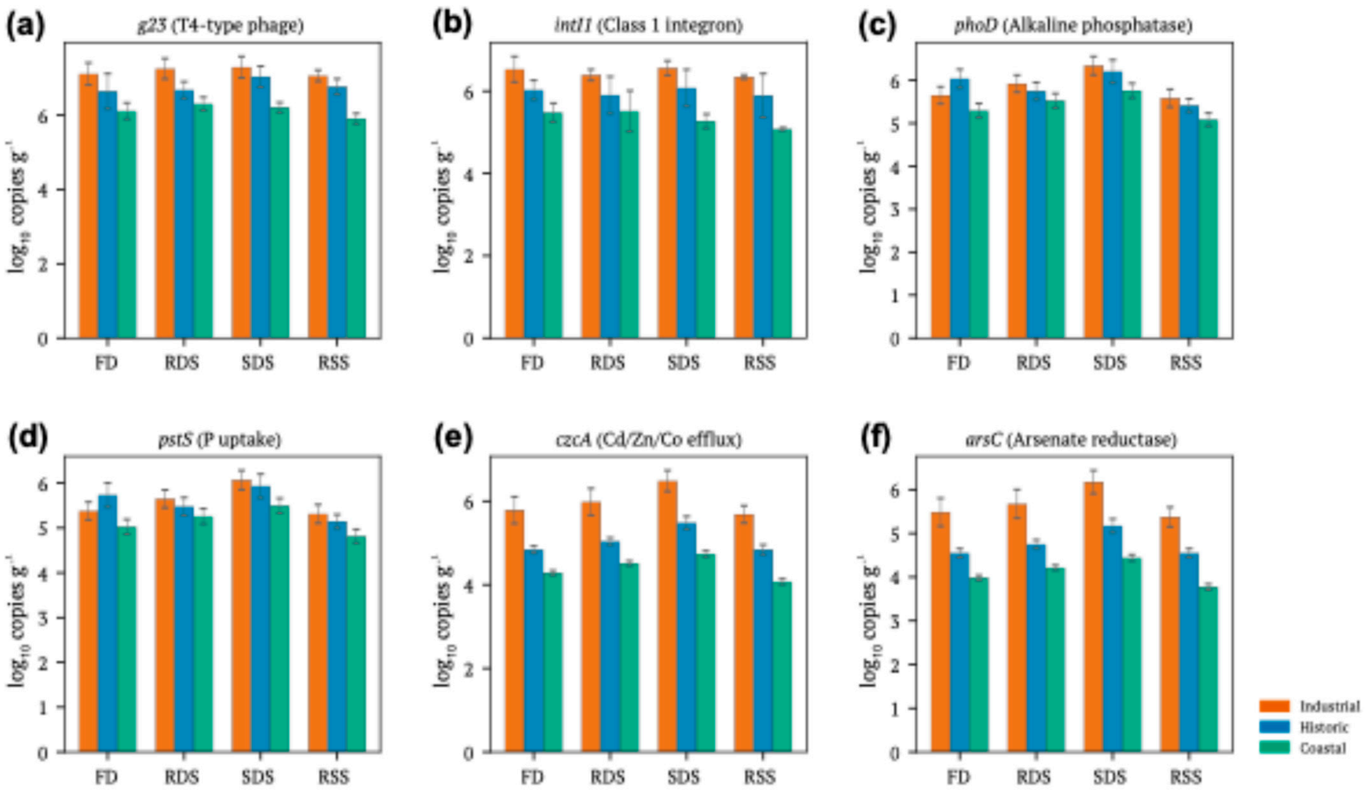

Figure S4. Functional gene abundances across sample types and functional zones. Abundance of (a) g23 (T4-type bacteriophage), (b) *intI1* (class 1 integron integrase), (c) *phoD* (alkaline phosphatase), (d) *pstS* (phosphate uptake), (e) *czcA* (Cd/Zn/Co efflux pump), and (f) *arsC* (arsenate reductase) genes across sample types, grouped by functional zone. All values are expressed as $\log_{10}$ copies g^{-1} dry weight. Error bars represent standard deviation (n = 3). Different colors represent functional zone types: Industrial (orange), Historic (blue), and Coastal (green).

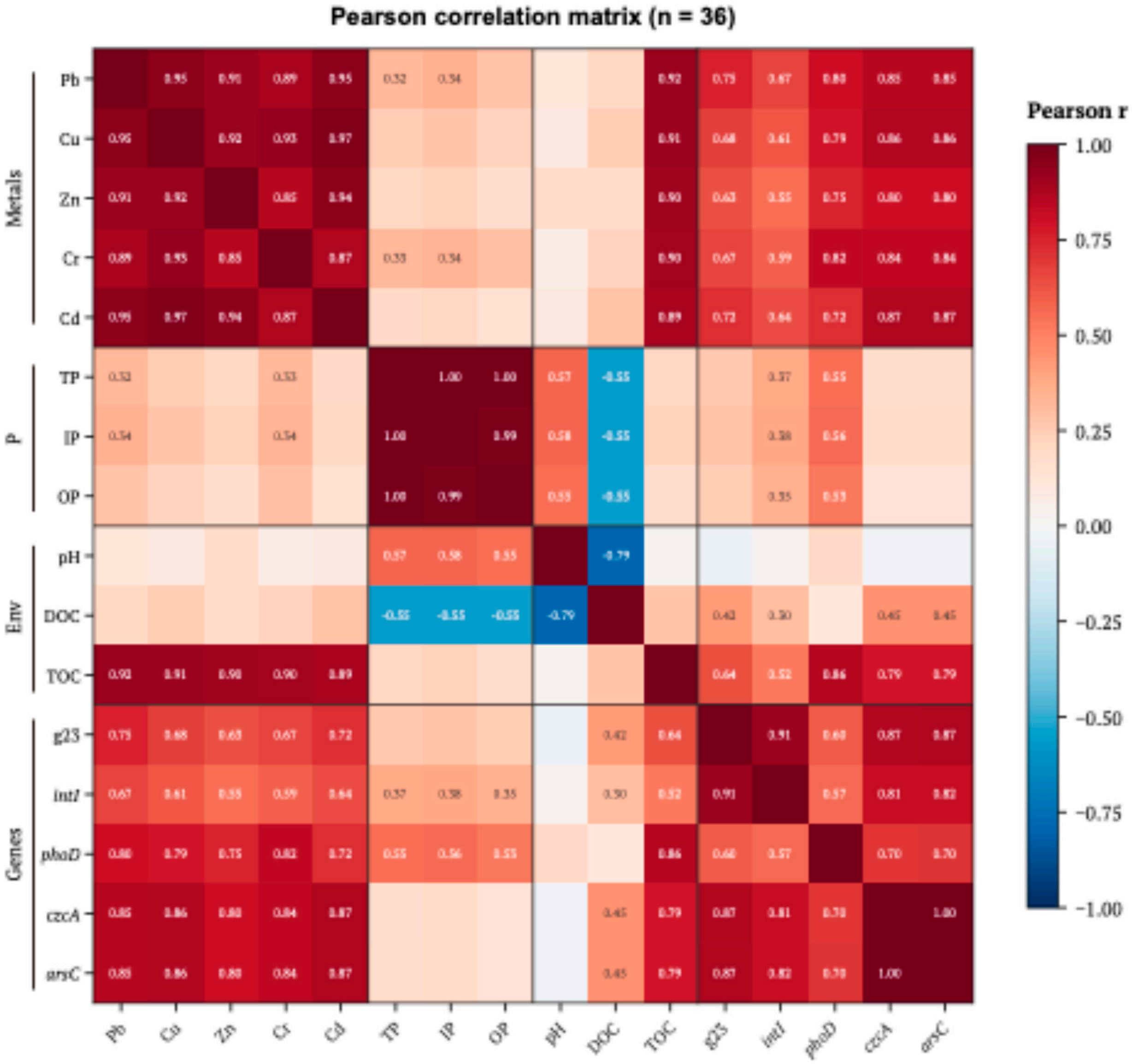

Figure S5. Pearson correlation matrix of heavy metals, phosphorus fractions, environmental parameters, and functional genes. Correlation coefficients are displayed for variable pairs with |r| > 0.30. The color scale indicates the strength and direction of correlations (red, positive; blue, negative). Variables are grouped into four categories: Metals (Pb, Cu, Zn, Cr, Cd), Phosphorus (TP, IP, OP), Environmental parameters (pH, DOC, TOC), and Genes (g23, *intI*, *phoD*, *czcA*, *arsC*). Black lines separate variable groups. n = 36 samples.

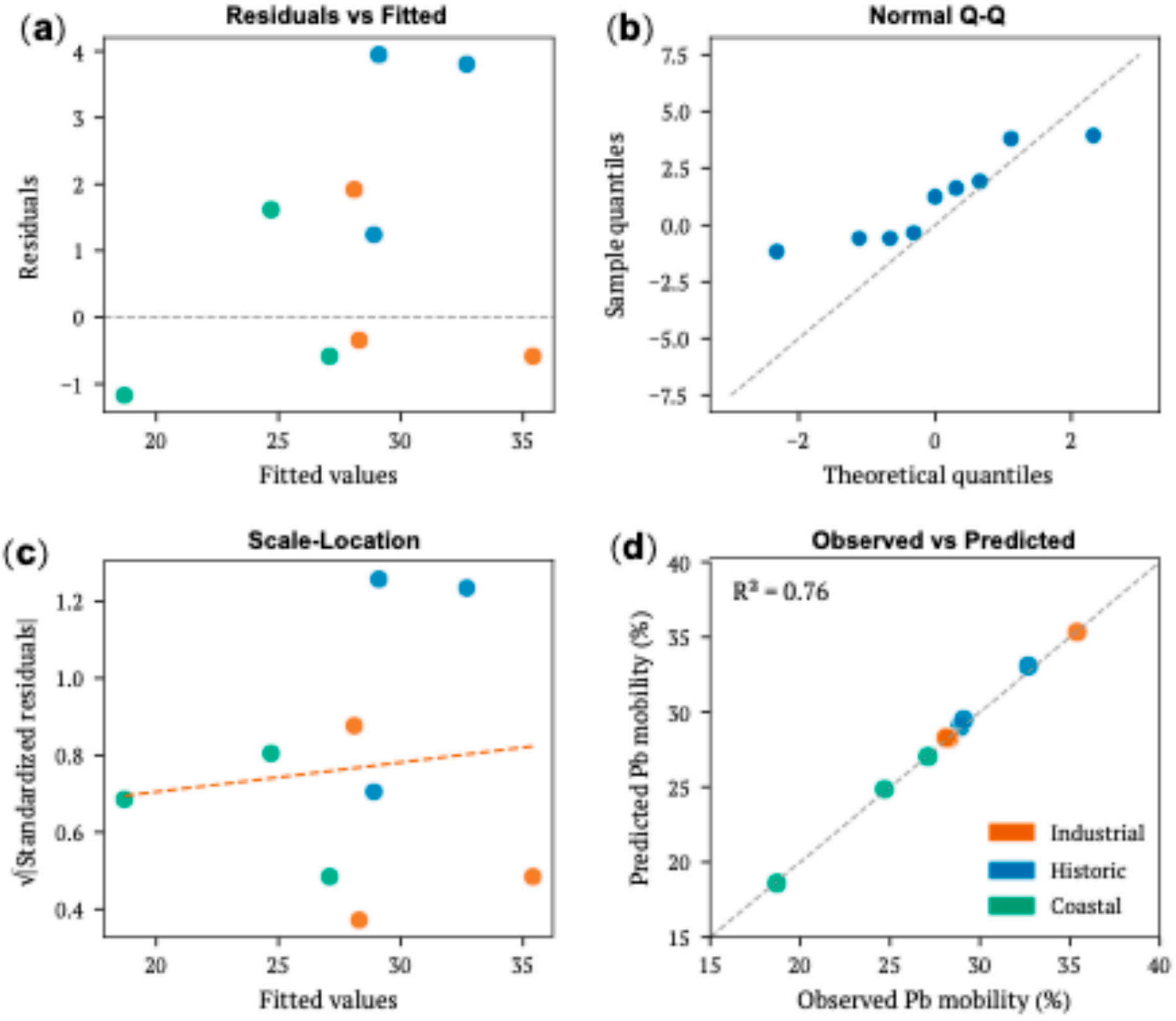

Figure S6. Diagnostic plots for the PLS path model. (a) Residuals versus fitted values plot for Pb mobility prediction. (b) Normal Q-Q plot of standardized residuals. (c) Scale-location plot showing the spread of residuals across fitted values. The dashed line shows the lowess smoothing trend. (d) Observed versus predicted Pb mobility values. The dashed diagonal line represents perfect prediction (1:1 line). Different colors represent functional zone types: Industrial (orange), Historic (blue), and Coastal (green). R2 = 0.76 indicates that the model explains 76% of the variance in observed Pb mobility.

https://doi.org/10.3390/toxics14030197

Table S1. BCR sequential extraction protocol for metal fractionation.

Fraction	Extractant	Conditions	Target Phase
F1 (Exchangeable)	0.11 M CH_3COOH	16 h, 22C, 30 rpm	Water-soluble and exchangeable
F2 (Reducible)	0.5 M NH_2OH-HCl (pH 1.5)	16 h, 22C, 30 rpm	Fe/Mn oxides bound
F3 (Oxidizable)	H_2O_2 8.8 M + 1 M NH_4OAc	1 h 85C, then 16 h 22C	Organic matter and sulfides
F4 (Residual)	HNO_3-HF-$HClO_4$ digestion	Microwave, 180C	Silicate lattice bound

Note: Modified from Rauret et al. (1999). Solid:liquid ratio = 1:40. All extractions performed in triplicate.

Table S2. Primer sequences and PCR conditions for qPCR analysis.

Target Gene	Function	Forward Primer (5'-3')	Reverse Primer (5'-3')	Amplicon (bp)	Reference
g23	T4-type phage capsid	AATGGTMCGTCTTGATGAY	ACMCCRGAYAARCCRGCAGC	450	Filee et al., 2005 [1]
intI1	Class 1 integron integrase	CCTCCCGCACGATGATC	TCCACGCATCGTCAGGC	280	Goldstein et al., 2001 [2]
phoD	Alkaline phosphatase	TGGGAYGATCAYGARGT	CTGSGCSAKSACRTTCCA	320	Ragot et al., 2015 [3]
pstS	Phosphate ABC transporter	TGCTSAAYCCSGCSATCGA	GCSGGRTCYTTSACGCC	285	Zheng et al., 2017 [4]
czcA	Cd/Zn/Co efflux pump	TGTTCAACCTGATGCTGCC	ATCGCCCACATGCTGAA	380	Nies, 1999 [5]
arsC	Arsenate reductase	GTSGGBTGYGGNAARAC	CARYCACGWGGATATCGAT	340	Sun et al., 2004 [6]
16S rRNA	Bacterial abundance	CCTACGGGAGGCAGCAG	ATTACCGCGGCTGCTGG	194	Muyzer et al., 1993 [7]

https://doi.org/10.3390/toxics14030197

Table S3. Pearson correlation matrix for metal mobility and environmental variables in SDS (n = 9).

	**Pb Mob.**	**Cu Mob.**	**Zn Mob.**	**pH**	**TP**	**g23**	***intI***
Pb Mob.	1.00						
Cu Mob.	0.68**	1.00					
Zn Mob.	0.72**	0.65**	1.00				
pH	-0.71**	-0.58*	-0.62**	1.00			
TP	0.48*	0.42	0.55*	-0.35	1.00		
g23	0.52**	0.45*	0.61**	-0.38	0.28	1.00	
intI	0.44*	0.52*	0.48*	-0.42	0.35	0.58**	1.00

Note: Mobility = F1 + F2 fraction (%). *p < 0.05, **p < 0.01. Lower triangle shows correlations; upper triangle omitted for clarity.

Table S4. Tukey HSD multiple comparison results for significant ANOVA effects.

Variable	Comparison	Mean Diff.	95% CI	Adj. p
Pb	SDS vs FD	+95.2	[68.5, 121.9]	<0.001***
Pb	SDS vs RDS	+57.8	[31.1, 84.5]	<0.001***
Pb	SDS vs RSS	+137.4	[110.7, 164.1]	<0.001***
Cu	SDS vs FD	+60.8	[42.3, 79.3]	<0.001***
Cu	SDS vs RDS	+45.2	[26.7, 63.7]	<0.001***
Zn	SDS vs FD	+277.4	[198.2, 356.6]	<0.001***
Zn	SDS vs RDS	+141.8	[62.6, 221.0]	<0.001***
TP	FD vs SDS	+523.8	[312.4, 735.2]	<0.001***
TP	FD vs RSS	+783.5	[572.1, 994.9]	<0.001***

Note: Only comparisons with adjusted $p < 0.05$ are shown. CI = confidence interval. ***$p < 0.001$.

References

1. Filée, J.; Tétart, F.; Suttle, C.A.; Krisch, H.M. Marine T4-type bacteriophages, a ubiquitous component of the dark matter of the biosphere. Proc. *Natl. Acad. Sci. U. S. A.* **2005**, *102*, 12471–12476. https://doi.org/10.1073/pnas.0503404102. PMID: 16116082.

2. Goldstein, C.; Lee, M.D.; Sanchez, S.; Hudson, C.R.; Phillips, B.; Register, B.; Grady, M.; Liebert, C.A.; Summers, A.O.; White, D.G.; Maurer, J.J. Incidence of Class 1 and 2 Integrases in Clinical and Commensal Bacteria from Livestock, Companion Animals, and Exotics. *Antimicrob. Agents Chemother* **2001**, *45*, 723–726. https://doi.org/10.1128/aac.45.3.723-726.2001. PMID: 11181350.
3. Ragot, S.A.; Kertesz, M.A.; Bünemann, E.K. phoD Alkaline Phosphatase Gene Diversity in Soil. *Appl. Environ. Microbiol.* **2015**, *81*, 7281–7289. https://doi.org/10.1128/AEM.01823-15. PMID: 26253682.
4. Zheng, J.-X.; Wu, Y.; Lin, Z.-W.; Pu, Z.-Y.; Yao, W.-M.; Chen, Z.; Li, D.-Y.; Deng, Q.-W.; Qu, D.; Yu, Z.-J. Characteristics of and Virulence Factors Associated with Biofilm Formation in Clinical Enterococcus faecalis Isolates in China. *Front Microbiol* **2017**, *8*, 2338. https://doi.org/10.3389/fmicb.2017.02338. PMID:29225595.
5. Nies, D.H. Microbial heavy-metal resistance. *Appl. Microbiol. Biotechnol.* **1999**, *51*, 730–750. https://doi.org/10.1007/s002530051457. PMID: 10422221.
6. Sun, Y.; Polishchuk, E.A.; Radoja, U.; Cullen, W.R. Identification and quantification of arsC genes in environmental samples by using real-time PCR. *J. Microbiol. Methods* **2004**, *58*, 335–349. https://doi.org/10.1016/j.mimet.2004.04.015. PMID: 15279938.
7. Muyzer, G.; de Waal, E.C.; Uitterlinden, A.G. Profiling of complex microbial populations by denaturing gradient gel electrophoresis analysis of polymerase chain reaction-amplified genes coding for 16S rRNA. *Appl. Environ. Microbiol.* **1993**, *59*, 695–700. https://doi.org/10.1128/aem.59.3.695-700.1993. PMID: 7683183.
